# Using AberOWL for fast and scalable reasoning over BioPortal ontologies

**DOI:** 10.1186/s13326-016-0090-0

**Published:** 2016-08-08

**Authors:** Luke Slater, Georgios V. Gkoutos, Paul N. Schofield, Robert Hoehndorf

**Affiliations:** 1 College of Medical and Dental Sciences, Institute of Cancer and Genomic Sciences, Centre for Computational Biology, University of Birmingham, Birmingham, B15 2TT United Kingdom; 2 Institute of Translational Medicine, University Hospitals Birmingham NHS Foundation Trust, Birmingham, B15 2TT United Kingdom; 3 Department of Physiology, Development and Neuroscience, University of Cambridge, Downing Street, England, CB2 3EG United Kingdom; 4 Computational Bioscience Research Center, Computer, Electrical and Mathematical Sciences & Engineering Division, King Abdullah University of Science and Technology, 4700 KAUST, Thuwal, 23955-6900 Saudi Arabia

**Keywords:** Ontology, Reasoning, Scalable reasoning, Description logics, OWL, AberOWL

## Abstract

**Background:**

Reasoning over biomedical ontologies using their OWL semantics has traditionally been a challenging task due to the high theoretical complexity of OWL-based automated reasoning. As a consequence, ontology repositories, as well as most other tools utilizing ontologies, either provide access to ontologies without use of automated reasoning, or limit the number of ontologies for which automated reasoning-based access is provided.

**Methods:**

We apply the AberOWL infrastructure to provide automated reasoning-based access to all accessible and consistent ontologies in BioPortal (368 ontologies). We perform an extensive performance evaluation to determine query times, both for queries of different complexity and for queries that are performed in parallel over the ontologies.

**Results and conclusions:**

We demonstrate that, with the exception of a few ontologies, even complex and parallel queries can now be answered in milliseconds, therefore allowing automated reasoning to be used on a large scale, to run in parallel, and with rapid response times.

## Background

Major ontology repositories such as BioPortal [1], OntoBee [2], or the Ontology Lookup Service [3], have existed for a number of years, and currently contain several hundred ontologies. They enable ontology creators and maintainers to publish their ontology releases and make them available to the wider community.

Besides the hosting functionality that such repositories offer, they usually also provide certain web-based features for browsing, comparing, visualising and processing ontologies. One particularly useful feature, currently missing from the major ontology repositories, is the ability to provide online access to reasoning services simultaneously over many ontologies. Such a feature would enable the use of semantics and deductive inference when processing data characterized by the ontologies these repositories contain [4].

For example, there is an increasing amount of RDF [5] data becoming available through public SPARQL [6] endpoints [7–10], which utilise ontologies to annotate entities, and access to reasoning over ontologies will allow combined queries over knowledge contained in ontologies and the data accessible through the SPARQL endpoints [4].

However, enabling automated reasoning over multiple ontologies is a challenging task, since automated reasoning can be highly complex and costly in terms of time and memory consumption [11]. In particular, ontologies formulated using the Web Ontology Language (OWL) [12] can utilize statements based on highly expressive description logics [13], and therefore queries that utilize automated reasoning cannot, in general, be guaranteed to finish in a reasonable amount of time.

Prior work on large-scale automated reasoning over biomedical ontologies has often focused on the set of ontologies in Bioportal, as it is one of the largest collections of ontologies freely available. To enable inferences over this set of ontologies, modularization techniques have been applied [14] using the notion of locality-based modules, and demonstrated that, for most ontologies and applications, relatively small modules can be extracted over which queries can be answered more efficiently. Other work has focused on predicting the performance of reasoners when applied to the set of BioPortal ontologies [15], and have demonstrated that performance of particular reasoners can reliably be predicted; at the same time, the authors have conducted an extensive evaluation of the average *classification* time for each ontology. Further approaches apply RDFS reasoning [16] to provide limited, yet fast, inference capabilities in answering queries over Bioportal’s set of ontologies through a SPARQL interface [17, 18]. Alternatively, systems such as OntoQuery [19] provide web-based access to ontologies through automated reasoning but limit the number of ontologies. The performance of OntoQuery has been found to be comparable to the performance of reasoning over ontologies in tools such as Protege [19].

The AberOWL [4] system is an ontology repository which aims to allow access to multiple ontologies through automated reasoning, utilizing their OWL semantics. AberOWL mitigates the complexity challenges by using a reasoner which supports only a subset of OWL (i.e., the OWL EL profile [20]), ignoring ontology axioms and queries that do not fall within this subset. This enables the provision of polynomial-time reasoning, which is sufficiently fast for many practical uses, even when applied to large ontologies [21]. However, thus far, the AberOWL software has only been applied to a few, manually selected ontologies, and therefore does not have a similar domain coverage to other ontology repositories, nor does it cater for reasoning over large sets of ontologies, such as the ones provided by the BioPortal ontology dataset (Bioportal contains, as of 9 March 2015, 428 ontologies consisting of 6,668,991 classes).

Here, we apply the AberOWL framework to reason over the majority of the ontologies available in Bioportal. We evaluate the performance of querying ontologies with AberOWL, utilizing 337 ontologies from BioPortal. We evaluate AberOWL’s ability to perform different types of queries as well as assess its scalability in performing queries that are executed in parallel. We demonstrate that the AberOWL framework makes it possible to provide, at least, light-weight description logic reasoning over most of the freely accessible ontologies contained in BioPortal, with a relatively low memory footprint and high scalability with respect to the number of queries executed in parallel, using only a single medium-sized server as hardware to provide these services. Furthermore, we identify several ontologies for which the performance of reasoning-based queries is significantly worse than the majority of the other ontologies tested, and discuss potential explanations and solutions.

## Methods

### Selection of ontologies

We selected all ontologies contained in BioPortal as candidate ontologies, and attempted to download the current versions of all the ontologies for which a download link was provided by BioPortal. A summary of the results is presented in Table 1.

**Table 1:** Summary of Ontologies used in our test

Total	427
Loadable	368
Used	337
Unobtainable	39
Non-parseable	17
Inconsistent	3
No Labels	31

The loadable ontologies are the ones obtained from BioPortal which could be parsed using the OWL API and which were found to be consistent when classified with the ELK reasoner. We exclude 31 ontologies that do not contain any labels from our analysis

Out of a total of 427 ontologies listed by Bioportal, only 368 could be directly downloaded and processed by AberOWL. Reasons for failure to load ontologies include the absence of a download link for listed ontologies, ontologies that are only available in proprietary data formats (e.g., some of the ontologies and vocabularies are provided in custom representation languages as part of the Unified Medical Language Systems [22]), or license restrictions which prevents their inclusion in public ontology repositories (e.g., SNOMED CT). Thirty nine ontologies were not obtainable. Furthermore, 17 ontologies that could be downloaded were not parseable with the OWL API, indicating a problem in the file format used to distribute the ontology. Three ontologies were inconsistent at the reasoning stage. Whilst several ontologies also included unobtainable ontologies as imports, we included these ontologies in our analysis, utilizing only the classes and axioms that were accessible. As AberOWL currently relies on the use of labels to construct queries, we further removed 31 ontologies that did not include any class labels from our test set.

Overall, we use a set of 337 ontologies in our experiments consisting of 3,466,912 classes and 6,997,872 logical axioms (of which 12,721 are axioms involving relations, i.e., RBox axioms). In comparison, BioPortal currently (9 March 2015) includes a total of 6,668,991 classes.

### Use of the AberOWL reasoning infrastructure

AberOWL [4] is an ontology repository and query service built on the OWLAPI [23] library, which allows access to a number of ontologies through automated reasoning. In particular, AberOWL allows users or software applications to query a single ontology within AberOWL using Manchester OWL Syntax [24], using the class and property labels as short-form identifiers for classes. Multiple queries to single ontologies can be performed at the same time, and AberOWL also provides functionality to perform a query on all ontologies within the repository. AberOWL exposes this functionality over the Internet through a JSON API as well as a web interface available on http://aber-owl.net. To answer queries, AberOWL utilizes the ELK reasoner [25, 26], a highly optimized reasoner that supports the OWL-EL profile. Ontologies which are not OWL-EL are processed by the reasoner by means of ignoring all non-EL axioms, although as of 2013 50.7 % of ontologies in Bioportal were natively using OWL-EL [27].

We extended the AberOWL framework to obtain a list of ontologies from the Bioportal repository, periodically checking for new ontologies as well as for new versions of existing ontologies. As a result, our testing version of AberOWL maintains a mirror of the accessible ontologies available in BioPortal. Furthermore, similarly to the functionality provided by BioPortal, a record of older versions of ontologies is kept within AberOWL, so that, in the future, the semantic difference between ontology versions can be computed.

In addition, we expanded the AberOWL software to count and provide statistics about:

- the ontologies which failed to load, with associated error messages;
- axioms, axiom types, and number of classes per ontology; and
- axioms, axiom types, and number of classes over all ontologies contained within AberOWL.

For each query of AberOWL, we also record the query execution time within AberOWL and pass this information back to the client along with the result-set of the query. Thus, the figures presented here do not include the time required to transmit the response.

All information is available through AberOWL’s JSON API http://aber-owl.net/help, and the source code is freely available at https://github.com/bio-ontology-research-group/AberOWL.

### Experimental setup

In order to evaluate the performance of querying single and multiple ontologies in AberOWL, queries of different complexity were randomly generated and executed. Since the ELK reasoner utilises a cache for answering queries that have already been computed, each of the generated queries consisted of a new class expression. The following types of class expressions were used in the generated queries (for randomly generated class labels A, B, and relation R):

- Primitive class: A
- Conjunctive query: A and B
- Existential query: R some A
- Conjunctive existential query: A and R some B

Three hundred random queries for each of these types were generated for each ontology that was tested (1200 queries in total per ontology). Each set of the 300 random queries generated, were subsequently split into three sets, each of which contained 100 class expressions. The random class expressions contained in the resulting sets were then utilised to perform superclass (100 queries), equivalent (100 queries) and subclass (100 queries) queries, and the response time of the AberOWL framework was recorded for each of the queries.

We further test the scalability of answering the queries by performing the queries in parallel. For this purpose, we perform three separate tests, to query AberOWL with one query at once, 100 queries in parallel, and 1,000 queries in parallel.

In our test, we record the response time of each query, based on the statistics provided by the AberOWL server; in particular, response time does not include network latency. All tests are performed on a server with 128 GB memory and two Intel Xeon E5-2680v2 10-core 2.8 GHz CPUs with hyper-threading activated (resulting in 40 virtual cores). The ELK reasoner underlying AberOWL is permitted to use all available (i.e., all 40) cores to perform classification and respond to each query.

## Results and discussion

On average, when performing a single query over AberOWL, query results are returned in 10.8 ms (standard deviation: 48.0 ms). The time required to answer a query using AberOWL correlates linearly with the number of logical axioms in the ontologies (Pearson correlation, *ρ*=0.33), and also strongly correlates with the number of queries performed in parallel (Pearson correlation, *ρ*=0.82).

**Fig. 1:**
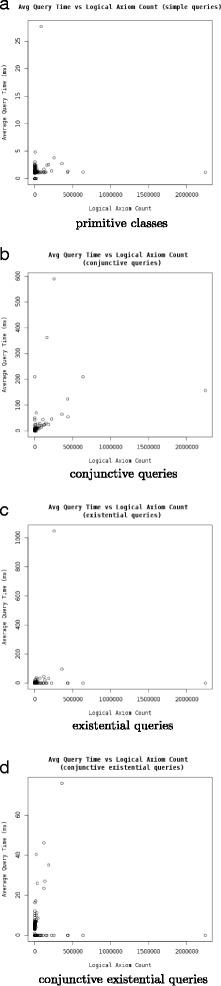
Query times as a function of the number of logical axioms in the ontologies, separated by the type of query

**Fig. 2:**
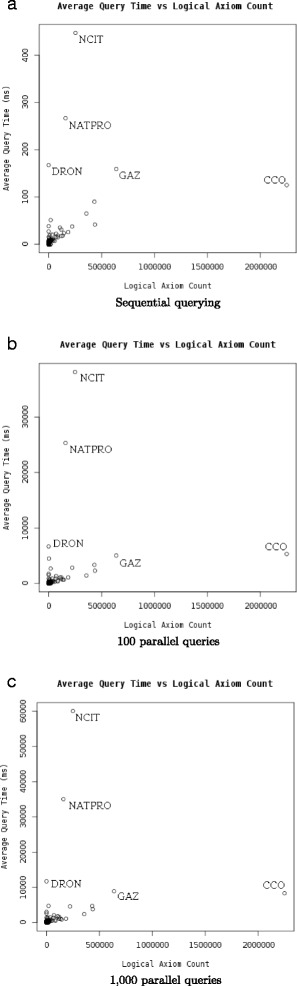
Query times as function of the number of logical axioms in the ontologies, separated by the number of queries executed in parallel

Figure 1 shows the query times for the ontologies based on the type of query, and Fig. 2 shows the query times based on different number of queries run in parallel. The maximum observed memory consumption for the AberOWL server while performing these tests was 66.1 GB.

**Fig. 3:**
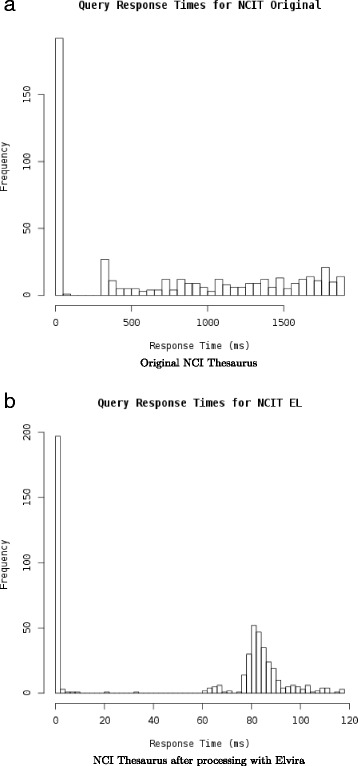
Query times over the NCI Thesaurus

We observe several ontologies for which query times are significantly higher than for the other ontologies. The most prevalent outlier is the NCI Thesaurus [28] for which average query time is 600 ms when performing a single query over AberOWL. Previous analysis of NCI Thesaurus has identified axioms which heavily impact the performance of classification for the ontology using multiple description logic reasoners [29]. The same analysis has also shown that it is possible to significantly improve reasoning time by adding inferred axioms to the ontology. To test whether this would also allow us to improve reasoning time over the NCI Thesaurus in AberOWL and using the ELK reasoner, we apply the Elvira modularization software [21], using the HermiT reasoner to classify the NCI Thesaurus to add all inferred axioms that fall into the OWL-EL profile to the ontology, as opposed to ELK’s approach of ignoring non-EL axioms during classification. We then repeat our experiments. Figure 3 shows the different reasoning times for NCI Thesaurus before and after processing with Elvira. Query time reduces from 703 ms (standard deviation: 689 ms) before processing with Elvira to 51 ms (standard deviation: 42 ms) after processing with Elvira, demonstrating that adding inferred axioms and removing axioms that do not fall in the OWL-EL profile can be used to improve query time.

Another outlier with regard to average query time is the Natural Products Ontology (NATPRO, http://bioportal.bioontology.org/ontologies/NATPRO). However, as NATPRO is expressed in OWL-Full, it cannot be reliably classified with a Description Logic reasoner, and therefore we could not apply the same approach to improve the performance of responding to queries.

### Future work

The performance of using automated reasoning for querying ontologies relies heavily on the type of reasoner used. We have used the ELK [25, 26] reasoner in our evaluation; however, it is possible to substitute ELK with any other OWLAPI-compatible reasoner. In particular, novel reasoners such as Konklude [30], which outperform ELK in many tasks [31], may provide further improvements in performance and scalability.

We identified several ontologies as leading to performance problems, i.e., they are outliers during query time testing. For these ontologies, including the Natural Products Ontology (NATPRO), and, to a lesser degree, the Drug Ontology (DRON) [32], similar ‘culprit-finding’ analysis methods may be applied as have previously been applied for the NCI Thesaurus [29]. These methods may also allow the ontology maintainers to identifying possible modifications to their ontologies that would result in better reasoner performance.

## Conclusion

We have demonstrated that it is feasible to reason over most of the ontologies available in BioPortal in real time, and that queries over these ontologies can be answered quickly, in real-time, and using only standard server hardware. We further tested the performance of answering queries in parallel, and show that, for the majority of cases, even highly parallel access allows quick response times which scale linearly with the number of queries.

We have also identified a number of ontologies for which performance of automated reasoning, at least when using AberOWL and the ELK reasoner, is significantly worse, which renders them particularly problematic for applications that carry heavy parallel loads. At least for some of these ontologies, pre-processing them using tools such as Elvira [21] can mitigate these problems.

The ability to reason over a very large number of ontologies, such as all the ontologies in BioPortal, opens up the possibility to frequently use reasoning not only locally when making changes to a single ontology, but also monitor – in real time – the consequences that a change may have on other ontologies, in particular on ontologies that may import the ontology which is being changed. Using automated reasoning over all ontologies within a domain therefore has the potential to increase interoperability between ontologies and associated data by verifying mutual consistency and enabling queries across multiple ontologies, and our results show that such a system can now be implemented with available software tools and commonly used server hardware.
